# Comparing Behavioral and Psychological Symptoms of Dementia and Caregiver Distress Caused Between Older Adults With Dementia Living in the Community and in Nursing Homes

**DOI:** 10.3389/fpsyt.2022.881215

**Published:** 2022-05-16

**Authors:** Xuejiao Lu, Rui Ye, Jialan Wu, Dongping Rao, Xiaoyan Liao

**Affiliations:** ^1^Department of Nursing, Nanfang Hospital, Southern Medical University, Guangzhou, China; ^2^Nursing School, Southern Medical University, Guangzhou, China; ^3^Geriatric Psychiatry, The Affiliated Brain Hospital of Guangzhou Medical University, Guangzhou, China

**Keywords:** community-dwelling, caregiver distress, dementia, BPSD, nursing home

## Abstract

**Objectives:**

To investigate differences in behavioral and psychological symptoms of dementia (BPSD) and caregiver distress caused between older adults with dementia living in the community and in nursing homes.

**Design:**

A comparative cross-sectional study.

**Setting and Participants:**

Participants were recruited from outpatient clinics of a tertiary psychiatric hospital and dementia units of a nursing home in Guangzhou, China.

**Methods:**

Neuropsychiatric Inventory was used to assess symptoms and caregiver distress. Dementia severity was determined using the Clinical Dementia Rating.

**Results:**

This study included 157 community and 112 nursing home residents with dementia. Clinically significant symptoms (item score ≥ 4) were found in 88.5% of the former and 75% of the latter. Caregivers of 79.6% of the former and 26.8% of the latter reported that at least one of these caused them moderate-to-severe distress (distress score ≥ 3). Among the community patients, anxiety was the most frequent “very severe” symptom, while sleep disorders and agitation caused the most frequent “very severe” caregiver distress. After controlling for dementia severity and medication use, family caregiving remained an independent risk predictor for clinically significant symptoms and moderate-to-severe caregiver distress. The prediction of caregiver distress based on symptom scores varied across caregiver types and individual symptoms (R^2^ 0.36–0.82). Group differences in clinically significant symptoms and moderate-to-severe caregiver distress showed at the stage of moderate-to-severe dementia.

**Conclusions and Implications:**

Tailored management strategies to relieve family caregivers’ BPSD-induced distress are needed, especially at the stage of moderate-to-severe dementia. An effective service system should be established for supporting family caregivers to cope with BPSD.

## Introduction

Dementia affects approximately 55.2 million people and their families worldwide in 2019, and this is expected to rise to 139 million by 2050 (1). Dementia care may be a long journey characterized by significant neuropsychiatric symptoms, caregiver distress, and unmet needs (2). Behavioral and psychological symptoms of dementia (BPSD) are common non-cognitive impairments occurring at all stages of dementia (3). Around 80%–90% of people with dementia have at least one of these symptoms (4, 5). One of the greatest challenges in caring for people with dementia is managing these symptoms. Difficulty in doing so often leads to higher caregiver burden (6), impaired quality of life (7, 8), increased healthcare utilization (9, 10), and risk of institutionalization (11). The problem is likely to be even greater within the community (12).

Family caregivers are mainly responsible for managing BPSD of people with dementia living at home (13–15), but typically possess only limited knowledge about symptoms and how to handle them (16). The number of people with dementia in China is estimated to exceed 9 million and is expected to reach 40 million by 2050 (17). Around 90% of them live at home and are looked after by their families (18). Dementia care services and related support services are also still developing in China (2). The lack of dementia-related training and community-based support services designed for people with dementia and their families can lead to exhaustion among family caregivers (18). Family caregivers in low- and middle-income countries often experience a higher level of distress due to the lack of resources and support mechanisms in the public healthcare system to help them properly managing BPSD (15, 19).

By comparison with their counterparts in long-term care facilities, we may get a better appreciation of clinically significant and moderate-to-severe distressing BPSD and their risk predictors in community-dwelling older adults with dementia cared by family caregivers. This is necessary to generate research evidence to inform the design of tailored management strategies and support family caregivers to enable people with dementia to stay at home longer, especially in low- and middle-income countries. Previous studies have investigated differences in caregiver distress caused by BPSD between community-dwelling older adults and residents of long-term care facilities (15, 20–22). Cheng and colleagues found that BPSD caused more distress to family caregivers than formal caregivers (20). On the contrary, Loi and colleagues suggest that formal caregivers may be exposed to more severe and frequent BPSD than informal caregivers (22). The discrepancies between the studies suggest that BPSD-induced distress may influence by a myriad of factors (such as country/culture, long-term care systems, outcome measures used, and sample characteristics). Especially, the use of samples with different severity of dementia, as well as a potential influence of medication used by participants, may lead to significant differences in the assessed severity of symptoms and the caregiver distress caused. We hypothesized that after controlling for dementia severity, as well as medication use, we might clarify the difference in BPSD and caregiver distress caused between community-dwelling older adults with dementia and nursing home residents with dementia.

This study therefore aimed to explore differences in BPSD and caregiver distress in responding to individual symptoms between community-dwelling older adults with dementia and their nursing home counterparts after controlling for dementia severity and medication use, and explore risk predictors of clinically significant symptoms and moderate-to-severe caregiver distress caused in the target population.

## Materials and Methods

### Study Design

A comparative cross-sectional study.

### Participants

Between March 2019 and September 2019, community-dwelling older adults with dementia and their primary family caregivers were recruited from the outpatient clinics of a 1920-bed tertiary psychiatric hospital in Guangzhou (the capital city of Guangdong province in China) using convenience sampling. The hospital provides neuropsychiatric services for 800 thousand outpatients per year. The inclusion criteria for community-dwelling older adults with dementia were (1) aged ≥ 60 years, lived at home and with support from family members; (2) had a physician diagnosis of dementia, following expert assessments. The exclusion criteria for older adults with dementia were (1) having past or present comorbidity of another major psychotic disorder, such as schizophrenia and bipolar disorders; (2) having a terminal illness (with life expectancy < 6 months); or (3) having significant variations in dosage of psychotropic medications during the past four weeks. The inclusion criteria for family caregivers were as follows: (1) the primary family caregiver of the patient; (2) had been in the role for at least 4 h per day during the past 3 months; (3) was able to read and speak Mandarin, and cognitively intact. The family caregiver who refused to participate was excluded. Nursing home residents with dementia were recruited from dementia units of nursing homes in Guangzhou, China. Eligibility for nursing home residents with dementia and their caregivers (the primary caregivers of the residents were formal caregivers who had the most day-to-day contact with the residents) has been described elsewhere (23).

Diagnosis of dementia was made using the Diagnostic and Statistical Manual of Mental Disorders, 4th edition (24). The diagnosis of Alzheimer’s disease (AD) was based on the NINCDS-ADRDA criteria (25), vascular dementia (VaD) was based on the NINDS-AIREN criteria (26), dementia with Lewy bodies (DLB) was based on DLB consensus criteria (27), and FTD was based on FTD consensus criteria (28).

### Measurement

#### Severity of Dementia

Severity of dementia was assessed using the Clinical Dementia Rating (CDR) (29). Clinical Dementia Rating is a global, informant-based, structured interview to determine the presence and severity of dementia. Information was collected from the persons with dementia and/or their informants about their performance in the following six domains: memory, orientation, judgment and problem-solving, community affairs, hobbies, and personal care. Each domain is rated on a 5-point scale (0 = none, 0.5 = questionable, 1 = mild, 2 = moderate, 3 = severe). A global rating of 1 (mild dementia), 2 (moderate dementia), and 3 (severe dementia) were used.

#### Behavioral and Psychological Symptoms of Dementia and Caregiver Distress Caused by Them

The Neuropsychiatric Inventory (NPI) was used to interview family caregivers. The NPI-Nursing Home Version (NPI-NH) was used to interview formal caregivers enrolled from nursing homes (23). Both NPI and NPI-NH examine 12 sub-domains of behavior functioning: delusions, hallucinations, agitation/aggression, depression/dysphoria, anxiety, elation/euphoria, apathy/indifference, disinhibition, irritability/liability, aberrant motor behaviors, sleep, and appetite and eating disorders. The 12 items were registered as present or not present during the past 4 weeks. If present, they were scored by frequency (1–4), severity (1–3), and level of caregiver distress (0 = not distressing to 5 = extremely). A higher score indicated more severe symptoms and a higher level of caregiver distress. We used the established cut-off item score (frequency × severity) of ≥ 4 to indicate a clinically significant symptom (21, 30, 31).

#### Basic Activities of Daily Living

Basic activities of daily living (BADL) were measured using the Barthel index (BI). We used a BI score of 0–20 to indicate total dependency, 21–60 for severe dependence, 61–90 for moderate dependence, and 91–99 for slight dependence (32).

### Data Collection

Clinical Dementia Rating (CDR) was rated by psychiatrists who worked in the tertiary psychiatric hospital and the nursing home from where the participants were enrolled. Primary caregivers were asked to complete the NPI-NH, and demographic information about care recipients was also collected through face-to-face interviews with the caregivers. The interviewers were three graduate students majoring in nursing, who received training in data collection, including all procedures and content of the assessments. Clinical data were obtained from medical records. Data about the actual use of medications were also collected based on the patients’ medical records. Medication use was classified according to the Anatomical Therapeutic Chemical classification, including anti-dementia drugs, antipsychotics, anxiolytics, anticonvulsants, and antidepressants.

### Sample Size Calculation

The sample size was calculated using a formula for calculating sample size in a prevalence study (33). The allowable error was assumed to be 10%. The level of confidence was set at 95%. Previous studies report the prevalence of BPSD ranged from 50.1 to 99% in Chinese community-dwelling persons with dementia (15, 21), and from 63.6 to 92.9% in Chinese nursing home residents with dementia (4, 23). According to the lowest prevalence rate of BPSD in previous studies, the sample size of this study should be at least 96 cases at each group. Considering 15% invalid questionnaires, 110 samples were required for this study, and 157 samples were finally collected from a community and 112 from a nursing home.

### Statistical Analysis

Statistical analysis used SPSS 25.0 (IBM SPSS Statistics, IBM Corp., Armonk, NY). Continuous variables were described as mean and standard deviation (SD), and categorical variables as number (n) and percentage (%). Non-parametric analyses were used when the data showed a non-normal distribution. Between-group comparisons used the Chi-square test (with Fisher’s exact test for multiple analyses) for categorical variables and Mann–Whitney U tests for continuous variables. Linear regression was conducted with continuous caregiver distress scores as a dependent variable and NPI item scores as independent variables. The NPI item score was dichotomized into ≥ 4 and < 4 to identify clinically significant symptoms, and caregiver distress score was dichotomized into ≥ 3 and < 3 for moderate-to-severe caregiver distress. Multivariate logistic regression (forward: conditionally) was used to test whether the odds of a clinically significant symptom and moderate-to-severe caregiver distress differed by CDR, caregiver type (family versus formal), medication use (anxiolytics, anticonvulsants, anti-depressants, anti-dementia, and antipsychotics), physical dependence, age, sex, and education. An additional independent variable for the latter was clinical significance of the symptoms (item score ≥ 4). As for controlling for the medication use, first, we compared medication use between the community-dwelling older adults with dementia and nursing home residents with dementia. And then, the multivariate logistic regressions above-mentioned were used in the full sample. A *P* value of less than 0.05 was considered statistically significant.

### Ethical Considerations

The Ethics Committee of N Hospital (No. NFEC-201511-K2) approved this study. Participants or their legal representatives were given a written and verbal explanation about the study, the benefits, and any risks. Written informed consent was obtained from all participants or their legal representatives. All personal information was kept confidential and anonymous as required.

## Results

The study included 157 community-dwelling older adults with dementia [age: 76.8 ± 9.9 (range 60–94) years; female: 93 (59.2%)] and 112 nursing home residents with dementia [age: 81.2 ± 8.1 (range 60–97) years; female: 41 (36.6%)]. Of those living in the community, 71 (45.5%) were cared for by their children, 64 (40.8%) by their spouse, and 22 (14%) by other relatives. The family caregivers spent an average of 12.2 ± 6.3 h per day providing supervision and caregiving. The demographic and clinical characteristics of the participants are shown in Table 1. The community patients were younger, with higher BI scores, and less severe dementia than the nursing home patients. They were also more likely to use anxiolytics, anti-depressants, and anticonvulsants (Table 1).

**TABLE 1:** Demographic and clinical characteristics of the participants.

Variables	Community (*n* = 157)	Nursing home (*n* = 112)
Age (years), Mean ± SD	76.8 ± 9.9***	81.2 ± 8.1
Female, n (%)	93 (59.2) ***	41(36.6)
Education, n (%)		
No schooling	14 (8.9)	19 (17.0)
Elementary school	72 (45.9)	35 (31.3)
Junior high school	30 (19.1)	28 (25.0)
Senior high school	32 (20.4)	16 (14.3)
University degree and above, n (%)	9 (5.7)	8 (7.1)
Missing data, n (%)	0 (0)	6 (5.4)
Diagnosis, n (%)		
AD	90 (57.3)	36 (32.1)
VaD	26 (16.6)	9 (8.0)
MD	13 (8.3)	5 (4.5)
DLB	3 (1.9)	0 (0)
FTD	2 (1.3)	1 (0.9)
Unknown	23 (14.6)	62 (55.4)
CDR, n (%) *		
CDR = 1	36 (22.9)	12 (10.7)
CDR = 2	47(29.9)	44 (39.3)
CDR = 3	74 (47.1)	56 (50.0)
Medication use, n (%)		
Anxiolytics	27 (17.2) ***	1 (0.9)^#^
Anti-depressants	21 (13.4) ***	1 (0.9)^#^
Anticonvulsants	13 (8.3)	5 (4.5)^#^
Anti-dementia	114 (89.1) ***	73 (65.2)
Antipsychotics	48 (49.1)	56 (37.5)
Missing data	29 (18.5)	0 (0)
BI, Mean ± SD	63.4 ± 33.1 ***	41.4 ± 32.0
NPI, Mean ± SD	41.0 ± 30.0 ***	16.0 ± 13.2
NPI disturbance, Mean ± SD	16.4 ± 13.3 ***	6.6 ± 6.4
Having at least one BPSDs, n (%)	142 (90.4)	104 (92.9)

*AD, Alzheimer’s disease; BI, Barthel Index; BPSD, Behavioral and psychological symptoms of dementia; CDR, Clinical Dementia Rating; DLB, dementia with Lewy Bodies. FTD, frontotemporal dementia; MD, mixed dementia, NPI, Neuropsychiatric Inventory; SD, standard deviation; VaD, Vascular dementia.*

*^#^ By using Fisher’s exact test.*

* P < 0.05, *** P < 0.001.

### Prevalence of Behavioral and Psychological Symptoms of Dementia

In total, 142 (90.4%) community patients and 104 (92.9%) nursing home patients had at least one symptom (*P* > 0.05). Apathy [community: 90 (57.4%); nursing home: 44 (39.3%)] was the most common symptom in both groups (Supplementary Table 1).

### Clinically Significant Behavioral and Psychological Symptoms of Dementia

The community patients had higher NPI item scores than the nursing home patients, except for euphoria, disinhibition, and eating disorders (Supplementary Table 1). Clinically significant symptoms were found in 139 (88.5%) community patients and 84 (75%) of the nursing home patients (χ^2^ = 8.45, *P* = 0.004).

Apathy was the most frequent clinically significant symptom in both the community (57.4%) and nursing home (39.3%) patients, followed by anxiety (47.8%), agitation (47.1%), and aberrant motor behaviors (47.1%) in the former, while aberrant motor behaviors (25.1%), irritability (24.1%), and agitation (20.6%) in the latter (Supplementary Table 2).

**FIGURE 1:**
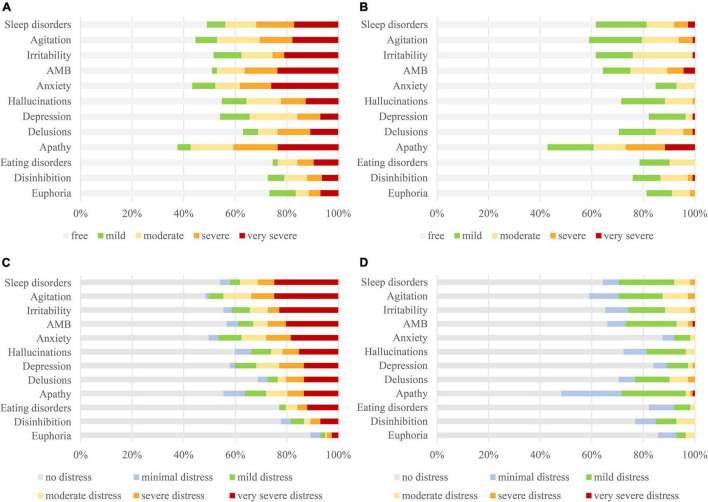
Comparing severity levels of BPSD [**(A)**: community; **(B)**: nursing home] and caregiver distress [**(C)**: community; **(D)**: nursing home] between community and nursing home residents with dementia. BPSD, behavioral and psychological symptoms of dementia.

Figure 1 shows that anxiety (26.1%) was the most frequent “very severe” symptom in the community patients, followed by apathy (23.6%), aberrant motor behaviors (23.6%), and irritability (21.0%). Apathy (11.6%) was the most frequent “very severe” symptom in the nursing home patients, followed by aberrant motor behaviors (4.5%) and sleep disorders (2.7%).

### Caregiver Distress Caused by Behavioral and Psychological Symptoms of Dementia

Overall, the caregivers of 125 (79.6%) community patients and 30 (26.8%) nursing home patients reported moderate-to-severe distress (distress score ≥ 3) caused by at least one symptom (χ^2^ = 74.72, *P* < 0.001). In total, 110 (70.1%) community patients and 11 (9.8%) nursing home patients had at least one symptom causing severe caregiver distress (distress score ≥ 4) (χ^2^ = 95.86, *P* < 0.001).

Agitation was the most frequent moderate-to-severe symptom in both the community (44.6%) and nursing home (12.5%) patients, followed by sleep disorders (38.2%), anxiety (37.6%), and irritability (34.4%) in the former, while irritability (11.6%), delusions (9.8%), and sleep disorders (8.0%) in the latter (Supplementary Table 2).

Figure 1 shows that sleep disorders (24.8%) and agitation (24.8%) were the most frequent cause of “very severe” distress among caregivers of community patients, followed by irritability (22.9%), aberrant motor behaviors (20.4%), and anxiety (18.5%). In contrast, only apathy (0.9%) and aberrant motor behaviors (0.9%) caused “very severe” distress among caregivers of the nursing home patients.

### Predictors for Clinical Significance of Behavioral and Psychological Symptoms of Dementia

Table 2 shows that, after controlling for dementia severity and medication use, family caregiving remained an independent risk predictor for presenting clinically significant symptoms (OR 2.11∼15.41), except for euphoria, disinhibition, and delusions.

**TABLE 2:** Odds ratios of presenting clinically significant symptoms and moderate-to-severe caregiver distress caused by the symptoms.

Dependent	Model 1: Clinical significance (item score ≥ 4)					Model 2: Moderate-to-severe caregiver distress (distress score ≥ 3)				
	
	Independent	OR	95%CI for OR		*P*	Independent	OR	95%CI for OR		*P*
			Lower	Upper				Lower	Upper	
Delusions	Anxiolytics	4.49	1.81	11.14	0.001	Delusion ≥ 4	74.04	26.65	205.68	< 0.0001
	Anticonvulsants	0.22	0.09	0.55	0.001					
	Age	1.04	1.01	1.08	0.021					
	BI (61–90)^a^	3.39	1.44	7.98	0.005					
Hallucinations	Family	5.01	2.52	9.93	< 0.0001	Family	5.77	1.82	18.29	0.003
	CDR	2.35	1.52	3.61	< 0.0001	Hallucination ≥ 4	21.83	9.14	52.15	< 0.0001
Agitation	Family	3.45	1.98	6.01	< 0.0001	Family	5.28	2.07	13.45	< 0.0001
	Anticonvulsants	1.001	1.000	1.002	0.011	Agitation ≥ 4	88.25	34.25	227.35	< 0.0001
Irritability	Family	2.11	1.21	3.67	0.008	Family	6.82	2.53	18.39	< 0.0001
	CDR	1.87	1.28	2.72	0.001	Irritability ≥ 4	157.93	48.49	514.35	< 0.0001
AMB	Family	2.94	1.71	5.05	< 0.0001	Family	5.91	2.26	15.43	< 0.0001
	CDR	1.62	1.14	2.30	0.007					
Anxiety	Family	14.21	6.36	31.76	< 0.0001	Family	11.66	2.45	55.58	0.002
	CDR	2.01	1.35	2.99	0.001	Anxiety ≥ 4	45.975	16.505	128.069	< 0.0001
Depression	Family	15.41	5.35	44.38	< 0.0001	Family	5.81	1.48	22.86	0.012
	CDR	1.56	1.02	2.37	0.015	Depression ≥ 4	46.47	18.49	116.80	< 0.0001
Apathy	Family	3.80	2.08	6.93	< 0.0001	Family	9.34	3.12	28.01	< 0.0001
	CDR	1.59	1.02	2.48	0.040	Apathy ≥ 4	31.96	7.46	137.11	< 0.0001
	BI (91–100)^a^	0.20	0.07	0.56	0.002					
	BI (61–90)^a^	0.34	0.15	0.77	0.010					
Disinhibition	/	/	/	/	/	Disinhibition ≥ 4	85.88	24.05	306.71	< 0.0001
Euphoria	Anxiolytics	1.001	1.000	1.002	0.033	Euphoria ≥ 4	53.58	10.66	269.46	0.002
	CDR	1.84	1.03	3.27	0.039	Sex	0.21	0.05	0.93	0.040
Sleep disorders	Family	3.86	2.15	6.94	< 0.0001	Family	6.44	2.42	17.11	< 0.0001
	CDR	1.87	1.29	2.71	0.001	Sleep disorders ≥ 4	81.99	29.08	231.16	< 0.0001
Eating disorders	Family	2.48	1.14	5.42	0.023	Family	18.16	3.40	97.01	0.001
	Anti-depressants	1.001	1.000	1.002	0.046	Eating disorders ≥ 4	151.52	38.56	595.44	< 0.0001
	CDR	1.84	1.12	3.05	0.017					

*AMB, aberrant motor behavior; BI, Barthel index; CDR, Clinical Dementia Rating. Dependent variable in Model 1: item score ≥ 4 or < 4; Dependent variable in Model 2: caregiver distress score ≥ 3 or < 3. The following variables were included as independent variables in both models: caregiver types (family versus formal), CDR, physical dependence (BI score of 91–100 indicate “slight” dependence, 61–90 indicate “moderate” dependence, 21–60 indicate “severe” dependence or 0–20 indicate “total” dependency), sex, education, age, and medication use (Anxiolytics, anticonvulsants, Anti-depressants, anti-dementia, and antipsychotics). Additional independent variable in Model 2: clinical significance of the symptom (item score ≥ 4).*

*^a^Reference is “BI (0–20)”.*

### Predictors for Caregiver Distress Caused by Behavioral and Psychological Symptoms of Dementia

After controlling for dementia severity, medication use, and symptom severity, family caregiving remained an independent risk predictor for moderate-to-severe caregiver distress caused by BPSD (OR 2.11∼18.16), especially eating disorders and anxiety (Table 2).

**FIGURE 2:**
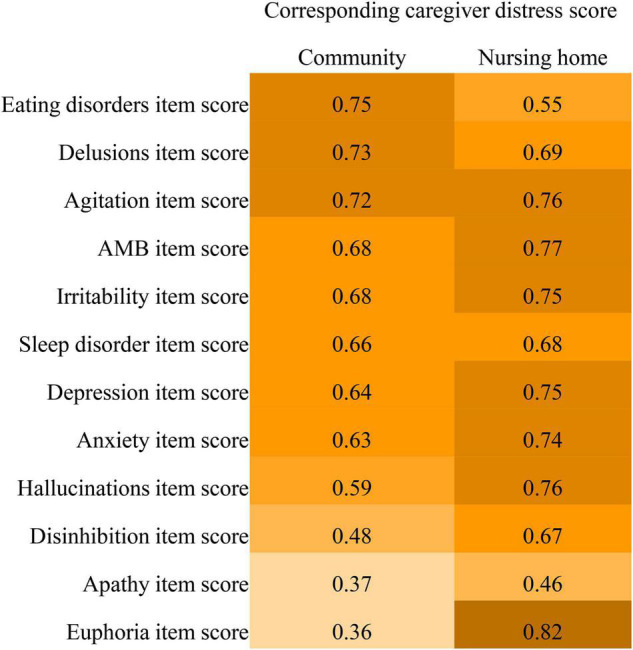
Comparisons of R^2^ values of the Neuropsychiatric Inventory item scores predicting caregiver distress between community-dwelling older adults with dementia and nursing home residents with dementia.

Linear regression analysis shows that caregiver distress was highly predicted by NPI item scores (*R*^2^ 0.63–0.82), except for apathy, euphoria, disinhibition, hallucination, and eating disorders (Figure 2). The apathy score did not highly predict caregiver distress for both the community (*R*^2^ = 0.37) and nursing home patients (*R*^2^ = 0.46). Item scores for euphoria (*R*^2^ = 0.36), disinhibition (*R*^2^ = 0.48), and hallucination (*R*^2^ = 0.59) did not highly predict caregiver distress in the community patients. Eating disorders scores did not highly predict caregiver distress in nursing home patients (R^2^ = 0.55).

### Comparisons Across Severity of Dementia

Among participants with mild dementia (CDR = 1), there were no group differences in prevalence, clinical significance, and moderate-to-severe caregiver distress caused by the symptoms (*P* > 0.05).

Among participants with moderate dementia (CDR = 2), anxiety, depression, and agitation were more clinically significant (anxiety: χ^2^ = 10.21, *P* = 0.001; depression: χ^2^ = 13.08, *P* < 0.001; agitation: χ^2^ = 4.88, *P* = 0.027) and moderate-to-severely distressing (anxiety: χ^2^ = 17.26, *P* < 0.001; depression: χ^2^ = 9.32, *P* = 0.002; agitation: χ^2^ = 5.39, *P* = 0.020) in community patients than their nursing home counterparts. However, there was no group difference in the prevalence of agitation (*P* > 0.05). Hallucinations (χ^2^ = 8.23, *P* = 0.004), apathy (χ^2^ = 6.16, *P* = 0.013), and aberrant motor behavior (χ^2^ = 4.67, *P* = 0.031) caused more frequent moderate-to-severe distress in the caregivers of community patients, but there was no group difference in the prevalence and clinical significance (*P* > 0.05).

**FIGURE 3:**
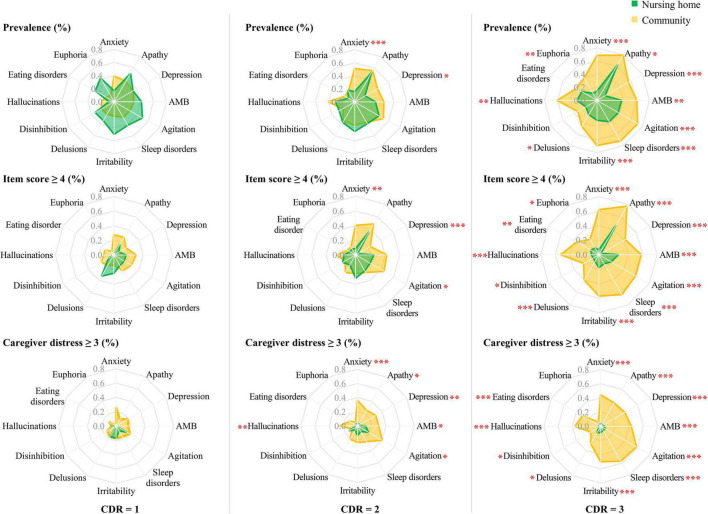
Comparisons of prevalence and clinical significance of BPSD (item score ≥ 4), and moderate-to-severe caregiver distress (distress score ≥ 3) caused by these symptoms, between community-dwelling older adults with dementia and nursing home residents with dementia, by severity of dementia. BPSD, behavioral and psychological symptoms of dementia; CDR, Clinical Dementia Rating. ^∗^ *P* < 0.05, ^∗∗^ *P* < 0.01, ^∗∗∗^ *P* < 0.001.

Among participants with severe dementia (CDR = 3), all symptoms except euphoria were more prevalent, clinically significant, and moderate-to-severely distressing in community patients than their nursing home counterparts (Figure 3). Euphoria was both more prevalent (χ^2^ = 11.34, *P* = 0.001) and more clinically significant (χ^2^ = 3.92, *P* = 0.048) in the community patients, but there was no difference in the distress it caused (*P* > 0.05). Eating disorders and disinhibition were more clinically significant (eating disorders: χ^2^ = 8.62, *P* = 0.003; disinhibition: χ^2^ = 5.19, *P* = 0.028) and moderate-to-severely distressing (eating disorders: χ^2^ = 15.23, *P* < 0.001; disinhibition: χ^2^ = 4.07, *P* = 0.044) in community patients, but there was no group difference in the prevalence of these symptoms (*P* > 0.05).

## Discussion

In this study, community-dwelling older adults with dementia showed more frequent clinically significant BPSD than nursing home patients, and these caused more severe caregiver distress. This was especially true for those with moderate-to-severe dementia. After controlling for dementia severity and medication use, family caregiving remained an independent risk predictor for presenting clinically significant symptoms and moderate-to-severe caregiver distress. The prediction of caregiver distress based on symptom severity varied across caregiver types. The strength of this study is that our findings were controlled for dementia severity and medication used by participants. Our findings add to the growing understanding of BPSD and caregiver distress they cause in low- and middle-income countries.

Clinically significant symptoms were found in 88.5% of community-dwelling older adults with dementia and 75% of nursing home patients in this study. After controlling for dementia severity and medication use, we found that caregiver type remained an independent risk predictor for presenting clinically significant BPSD. One potential explanation is that BPSD rely on caregiver proxies to report, and family caregivers may have more opportunities to observe these symptoms. Moreover, family caregivers might be more involved in affective disturbances of care recipients, and the internalizing symptoms of dementia, such as anxiety and depression, might be over-reported by family caregivers because of their increased risks for stress, depression, and burden. Another explanation is that formal caregivers are more likely to have had some training in managing BPSD (34). Training has been found to have an impact on the way staff behave toward residents with dementia (35). Family caregivers’ improper management of behavior problems might result in aggravation of the symptoms (36). Delivery appropriate training to caregivers has been proposed as a key component of good dementia care (34).

Impressively, up to 70% of the community-dwelling older adults with dementia had at least one symptom causing severe caregiver distress in this study. However, only 9.8% of the nursing home residents with dementia had at least one symptom causing severe caregiver distress. Family caregivers caring for community-dwelling older adults with dementia experienced more severe distress caused by symptoms than formal caregivers caring for nursing home residents with dementia. After controlling for symptom severity, family caregiving remaining an independent risk predictor for moderate-to-severe caregiver distress in this study, especially eating disorders and anxiety. One explanation is that family caregivers provide longer company for care recipients and undertake additional caregiving tasks than formal caregivers, while formal caregivers are more likely to work cooperatively to manage patients in their workplace than family caregivers. Another explanation is that there is currently insufficient quality and quantity of family based care services in China to meet the needs of community-dwelling older adults with dementia (2). Family caregivers may therefore experience a higher level of distress due to the lack of resources and support in the public health care system to manage and treat dementia symptoms (18).

Previous studies suggest that information on caregiver distress caused by individual symptoms is crucial to inform the design of tailored management strategies for supporting family caregivers (6, 15, 37). In this study, agitation and sleep disorders were the fifth and sixth frequent of “very severe” symptom but caused the most frequent “very severe” distress to family caregivers caring for community-dwelling older adults with dementia. However, the community-dwelling patients had a similar prevalence of agitation during the moderate stage of dementia, compared with their nursing home counterparts. Agitation is one of the most common symptoms across studies (6, 7, 37, 38). In the context of biopsychosocial approaches (36, 39, 40), agitation often occurs during personal care, and may be closely linked to expressions of unmet need (e.g., can be aggressive if attention is not given) and inadequate interactions between caregivers and care recipients (41). Sleep disorders in patients with dementia include fragmented sleep at night, daytime sleepiness, inversion of the sleep-wake cycle, and sleep behavior disorders (42). Various factors, such as the need to urinate during the night, brain stimulants (coffee and bronchodilators), pain, and medications (diuretics), may contribute to sleep disorders (43). Family caregivers have more exposure to care recipients at night (42, 44), and sleep disorders can therefore cause or exacerbate sleep deprivation and exhaustion in family caregivers. We also found that euphoria was more prevalent and clinically significant, but had no difference in the distress for caregivers of community patients with severe dementia, compared with the nursing home patients. Our findings should encourage healthcare providers to focus on clinically significant symptoms that produce severe distress in family caregivers, rather than those that are more prevalent. Similarly, a recent study suggests that clinicians should distinguish relatively untroubling symptoms from more exhausting symptoms (37).

In this study, community-dwelling older adults with dementia had more than ten times the risk of presenting clinically significant anxiety and depression, which causing five to ten times the risk of moderate-to-severe distress to family caregivers. Affective disturbances should be taken into account in alleviating distress for family caregivers. We also found that the severity of euphoria and disinhibition did not strongly predict caregiver distress caused in community-dwelling older adults, while the severity of eating disorders did not strongly predict caregiver distress caused in nursing home residents with dementia. Euphoria and disinhibition, which have been classified as “Frontal” endophenotype (45), were the rarest symptoms in both this study and previous studies (37, 45, 46). A recent study suggests that sexual disinhibition appears to be a particularly difficult symptom for the family caregiver (47). However, there were 125 (11.8%) FTD patients in that study, but only 2 (1.3%) FTD patients in this study. The low prevalence of euphoria and disinhibition might partially contribute to the low prediction of caregiver distress caused in this study. Formal caregivers may be more likely to cooperate with colleagues, and be less involved in providing daily care (such as support with eating). Therefore, eating disorders may not place such a physical and psychological burden on formal caregivers. Our findings suggest that intervention strategies for reducing caregiver distress caused by BPSD should consider caregiver types and individual symptoms.

From a public health perspective, optimizing dementia care in the home may provide the maximum population-level benefit (such as decreasing the huge economic costs associated with transitions to other care settings) and is desirable given the preference of older people to remain in their familiar communities for as long as possible (48). The majority of older people with dementia live at home with unpaid family caregivers, and these family caregivers are at risk for poor physical, mental, emotional, and socioeconomic outcomes (49). The present study found that family caregivers caring for community-dwelling older people with dementia were exposed to more frequent clinically significant and moderate-severe distressing BPSD than formal caregivers (nursing home staff). BPSD are the most treatable aspects of dementia and might be alleviated or eliminated through comprehensive interventions to target contributing factors or triggers (34). There are complex overlaps or interplay of reactive, responsive, and/or organic causes of BPSD (50). Caregiver factors are some of the causes and triggers of BPSD (36), for example, inadequate interactions between caregivers and care recipients may contribute to the occurrence or exacerbation of BPSDs. Reciprocally, exacerbation of BPSD may aggravate caregiver distress (15). Training and supporting family caregivers, especially for proper management of BPSD may help to break this vicious circle. Our findings suggest that an effective support service system should be established to give family caregivers access to resources, information, and knowledge to improve their ability to cope with BPSD. The support service system, such as Dementia Support programs proposed in Australia (51), might enable people with dementia to stay at home longer.

This study had several limitations. First, the cross-sectional design of this study does not allow for a test of causal relationships. Second, modest sample size and insufficient information on dementia subtypes limited further analysis. Third, our data were collected from family caregivers who voluntarily visited the outpatient clinic of a tertiary psychiatric hospital, which may have introduced referral bias. Fourth, the study did not investigate factors related to caregiver burden or mental health status (such as depression and anxiety). The above-mentioned factors might limit the generalizability of our findings. Future studies with a larger sample size should consider these factors.

## Conclusion and Implications

This study found that family caregivers experienced more severe distress caused by behavioral and psychological symptoms of dementia than formal caregivers; family caregiving was an independent risk predictor for clinically significant and moderate-to-severe distressing symptoms; the prediction of caregiver distress based on symptom scores varied by individual symptoms and caregiver types. Our findings highlight that tailored management strategies are needed to relieve family caregivers’ distress caused by BPSD, and that an effective support service system should be established to give family caregivers access to resources, information, and knowledge to improve their ability to cope with BPSD.

## Data Availability Statement

The original contributions presented in the study are included in the article/Supplementary Material, further inquiries can be directed to the corresponding author.

## Ethics Statement

The studies involving human participants were reviewed and approved by The Ethics Committee of Nanfang Hospital (No. NFEC-201511-K2) approved this study. The patients/participants provided their written informed consent to participate in this study.

## Author Contributions

XJL, JW, and RY gathered the data, completed the data analysis and interpretation, and took part in drafting of the manuscript. DR helped with acquisition of data and clinical diagnosis. XYL advised on the design of the study, secured funding, supervised the research, took responsibility for the accuracy of the data analysis and interpretation, and provided critical and substantive commentary to both the process and final product. All authors granted approval for submission and publication, contributed to revising the manuscript critically for important intellectual content, and were in agreement with the content of the manuscript.

## Conflict of Interest

The authors declare that the research was conducted in the absence of any commercial or financial relationships that could be construed as a potential conflict of interest.

## Publisher’s Note

All claims expressed in this article are solely those of the authors and do not necessarily represent those of their affiliated organizations, or those of the publisher, the editors and the reviewers. Any product that may be evaluated in this article, or claim that may be made by its manufacturer, is not guaranteed or endorsed by the publisher.
