# Supplementary material for: Comparing Behavioral and Psychological Symptoms of Dementia and Caregiver Distress Caused Between Older Adults With Dementia Living in the Community and in Nursing Homes
Source: Front Psychiatry. 2022 May 16;13:881215. doi: 10.3389/fpsyt.2022.881215 (PMC9150772; doi:10.3389/fpsyt.2022.881215)
Supplement: Supplementary file 1 [file Table_1.DOCX]

**Table S1**. Comparisons of prevalence, frequency × severity, and caregiver distress of BPSDs between community-dwelling older adults and nursing home residents.

| Item | Prevalence, n (%) | |  | Item score, mean ± SD | |  | Caregiver distress, mean ± SD | |
| --- | --- | --- | --- | --- | --- | --- | --- | --- |
|  | Community (n = 157) | NH (n = 112) |  | Community (n = 157) | NH (n = 112) |  | Community (n = 157) | NH (n = 112) |
| Delusions | 58 (36.9) | 34 (30.4) |  | 2.93 ± 4.41 * | 1.29 ± 2.46 |  | 1.16 ± 1.90 | 0.65 ± 1.12 |
| Hallucinations | 71 (45.2) ** | 31 (27.7) |  | 3.27 ± 4.44 *** | 0.91 ± 1.77 |  | 1.37 ± 1.94 ** | 0.50 ± 0.88 |
| Depression | 72 (45.9) *** | 20 (17.9) |  | 2.80 ± 3.82 *** | 0.46 ± 1.43 |  | 1.81 ± 2.04 *** | 0.22 ± 0.64 |
| Anxiety | 89 (56.7) *** | 17 (15.2) |  | 4.77 ± 5.12 *** | 0.46 ± 1.23 |  | 1.50 ± 1.95 *** | 0.30 ± 0.77 |
| Euphoria | 42 (26.8) | 21 (18.8) |  | 1.62 ± 3.46 | 0.69 ± 1.82 |  | 0.30 ± 1.02 | 0.25 ± 0.69 |
| Apathy | 98 (62.4) | 64 (57.1) |  | 5.03 ± 4.84 * | 3.57 ± 4.23 |  | 1.42 ± 1.88 | 0.87 ± 1.01 |
| Agitation | 87 (55.4) * | 46 (41.1) |  | 4.30 ± 4.70 *** | 1.73 ± 2.66 |  | 2.05 ± 2.16 *** | 0.86 ± 1.18 |
| Irritability | 76 (48.4) | 43 (38.4) |  | 3.83 ± 4.87 ** | 1.62 ± 2.47 |  | 1.71 ± 2.12 ** | 0.74 ± 1.14 |
| AMB | 77 (49.0) * | 40 (35.7) |  | 4.45 ± 5.07 *** | 1.94 ± 3.30 |  | 1.63 ± 2.09 ** | 0.71 ± 1.13 |
| Disinhibition | 43 (27.4) | 27 (24.1) |  | 1.83 ± 3.57 | 0.96 ± 2.14 |  | 0.72 ± 1.53 | 0.46 ± 0.92 |
| Sleep disorders | 80 (51.0) * | 43 (38.4) |  | 4.11 ± 4.77 *** | 1.74 ± 2.91 |  | 1.82 ± 2.18 ** | 0.75 ± 1.10 |
| Eating disorders | 40 (25.5) | 24 (21.4) |  | 2.08 ± 3.95 | 0.65 ± 1.53 |  | 0.94 ± 1.81 | 0.28 ± 0.66 |

Note: Item score = frequency × severity. AMB, Aberrant motor behavior; BPSD, Behavioral and Psychological Symptoms of Dementia; NH, nursing home; SD, standard deviation; Item score = frequency × severity.

* *P* < 0.05, ***P* < 0.01, *** *P* < 0.001.
