# Supplementary material for: Comparing Behavioral and Psychological Symptoms of Dementia and Caregiver Distress Caused Between Older Adults With Dementia Living in the Community and in Nursing Homes
Source: Front Psychiatry. 2022 May 16;13:881215. doi: 10.3389/fpsyt.2022.881215 (PMC9150772; doi:10.3389/fpsyt.2022.881215)
Supplement: Supplementary file 2 [file Table_2.DOCX]

**Table S2**. Comparisons of presenting clinically significant symptoms and caregiver distress between the community-dwelling older adults and the nursing home residents.

| Item | Clinical significance  (Item score ≥ 4; n%) | |  | Moderate-to-severe caregiver distress  (Caregiver distress score ≥ 3; n%) | |  | Severe caregiver distress  (Caregiver distress score ≥ 4; n%) | |
| --- | --- | --- | --- | --- | --- | --- | --- | --- |
|  | Community (n = 157) | NH (n = 112) |  | Community (n = 157) | NH (n = 112) |  | Community (n = 157) | NH (n = 112) |
| Delusions | 49 (31.1%) ** | 17 (15.2%) |  | 37 (23.6%) ** | 11 (9.8%) |  | 32 (20.4%) *** | 3 (2.7%) |
| Hallucinations | 56 (35.7%) *** | 13 (11.6%) |  | 41 (26.1%) *** | 4 (3.6%) |  | 34 (21.7%) *** | 0 (0.0%) |
| Depression | 54 (34.4%) *** | 4 (3.6%) |  | 50 (31.8%) *** | 3 (2.7%) |  | 36 (22.9%) *** | 1 (0.9%) |
| Anxiety | 75 (47.8%) *** | 8 (7.1%) |  | 59 (37.6%) *** | 2 (1.8%) |  | 44 (28.0%) *** | 0 (0.0%) |
| Euphoria | 26 (16.6%) | 10 (8.9%) |  | 8 (5.1%) | 4 (3.6%) |  | 7 (4.5%) * | 0 (0.0%) |
| Apathy | 90 (57.4%) ** | 44 (39.3%) |  | 44 (28.0%) *** | 4 (3.6%) |  | 31 (19.7%) *** | 2 (1.8%) |
| Agitation | 74 (47.1%) *** | 23 (20.6%) |  | 70 (44.6%) *** | 14 (12.5%) |  | 53 (33.8%) *** | 3 (2.7%) |
| Irritability | 59 (37.6%) * | 27 (24.1%) |  | 54 (34.4%) *** | 13 (11.6%) |  | 43 (27.4%) *** | 2 (1.8%) |
| AMB | 74 (47.1%) *** | 28 (25.1%) |  | 52 (32.1%) *** | 8 (7.1%) |  | 43 (27.4%) *** | 3 (2.7%) |
| Disinhibition | 33 (21.0%) | 15 (13.4%) |  | 21 (13.4%) | 8 (7.1%) |  | 17 (10.8%) *** | 0 (0.0%) |
| Sleep disorders | 50 (31.8%) *** | 9 (8.1%) |  | 60 (38.2%) *** | 9 (8.0%) |  | 49 (31.2%) *** | 2 (1.8%) |
| Eating disorders | 37 (23.6%) ** | 11 (9.8%) |  | 32 (20.4%) *** | 2 (1.8%) |  | 25 (15.9%) ** | 0 (0.0%) |

Note: Item score = frequency × severity. AMB, aberrant motor behavior; NH, nursing home.

* *P* < 0.05, ***P* < 0.01, *** *P* < 0.001.
